# Mitochondrial Genomic Landscape: A Portrait of the Mitochondrial Genome 40 Years after the First Complete Sequence

**DOI:** 10.3390/life11070663

**Published:** 2021-07-06

**Authors:** Alessandro Formaggioni, Andrea Luchetti, Federico Plazzi

**Affiliations:** Department of Biological, Geological and Environmental Sciences, University of Bologna, Via Selmi, 3, 40126 Bologna, BO, Italy; alessand.formaggioni@studio.unibo.it (A.F.); andrea.luchetti@unibo.it (A.L.)

**Keywords:** mitochondrial genome, mtDNA architecture, mtDNA structure, nucleotide composition, compositional bias, strand asymmetry, Eukaryota, mtDNA expansion

## Abstract

Notwithstanding the initial claims of general conservation, mitochondrial genomes are a largely heterogeneous set of organellar chromosomes which displays a bewildering diversity in terms of structure, architecture, gene content, and functionality. The mitochondrial genome is typically described as a single chromosome, yet many examples of multipartite genomes have been found (for example, among sponges and diplonemeans); the mitochondrial genome is typically depicted as circular, yet many linear genomes are known (for example, among jellyfish, alveolates, and apicomplexans); the chromosome is normally said to be “small”, yet there is a huge variation between the smallest and the largest known genomes (found, for example, in ctenophores and vascular plants, respectively); even the gene content is highly unconserved, ranging from the 13 oxidative phosphorylation-related enzymatic subunits encoded by animal mitochondria to the wider set of mitochondrial genes found in jakobids. In the present paper, we compile and describe a large database of 27,873 mitochondrial genomes currently available in GenBank, encompassing the whole eukaryotic domain. We discuss the major features of mitochondrial molecular diversity, with special reference to nucleotide composition and compositional biases; moreover, the database is made publicly available for future analyses on the MoZoo Lab GitHub page.

## 1. Introduction

Few myths in molecular biology are as stubbornly long-lived as the stability and conservation of mitochondrial genome (mtDNA) among animals (and eukaryotes), be it in terms of content, structure, or architecture. The first evidence that some animals harbor a covalently-closed mtDNA was provided in 1966 for chickens, cows, and mice [1,2]; in the very same years, a comparably small size was reported from a handful of animal groups [3]. As discussed in Williamson [4], this became the first, indisputable evidence for the intriguing hypothesis that was initially put forward by Altmann [5] about 80 years before: mitochondria are endosymbionts with a prokaryotic descent.

In this context, when linear DNA molecules were reported from unicellular eukaryotes [6], it was tempting to classify them as exceptions, and the “broken-circle theory” [7,8] was proposed for yeast mtDNA: if any linear mtDNA is observed in yeast, it ought to be a broken circle (see [4]). Moreover, this claim extended the supposed conservation of mtDNA to a different eukaryotic realm. The complete sequences of mtDNA from humans [9], mice [10], and cattle [11] were soon followed by the complete sequence from *Drosophila yakuba* [12]: genomes that were found to be (i) single, (ii) closed circles of (iii) comparable size, with (iv) a conserved genetic content. The myth of a “typical” mtDNA was born, at least for Metazoa [13].

Nevertheless, 40 years after the first complete mtDNAs, thousands of mtDNAs have been completely or partially sequenced, annotated, and compared, and it has become increasingly clear that these features are hardly conserved (if at all) among eukaryotes. In extreme cases, some eukaryotes did even lose mtDNA (or even organelles themselves; [13,14,15,16,17]).

(i) Multipartite genomes. Multipartite mtDNAs are, in fact, widespread among eukaryotes [15,18]. The mtDNA of *Trypanosoma brucei* (Euglenozoa: Kinetoplastea) is organized as a kinetoplast, a compact network of maxicircles (~25 kb) and thousands of minicircles (~1 kb), where mitochondrial genes and regulatory small RNAs are located, respectively ([18,19,20,21] and references therein). The mtDNA in other euglenozoans, Diplonemea, is composed of dozens of circular chromosomes; they can be subdivided into two size classes, with chromosomes of the same class sharing approximately 95% of the sequence. The remainder constitutes the only coding region of the chromosome, where one or more exons are located, ranging from 40 to 540 bp in length and relying on a complex trans-splicing and post-transcriptional machinery [19,22]. Mitochondrial genomes from Alveolata, and specifically of dinoflagellates, are also highly fragmented and possibly constitute the most divergent mitochondrial genomes among eukaryotes along with diplonemeans [23,24].

The structure of plant mtDNA is better understood as an entangled pattern where alternative molecules can coexist and recombine [7,15,25,26,27,28,29,30,31,32,33,34,35,36], while some mitochondria may contain only partial or no genome at all [37]. Occasionally, however, the mtDNA appears to be organized into stable, autonomous circles (e.g., [38,39]).

Among Opisthokonta, multiple mitochondrial chromosomes have been reported from Calcarea [13,40,41]; Hydrozoa and Cubozoa [42,43]; *Dicyema* [44]; Syndermata *sensu* Witek et al. [45,46,47]; Nematoda [48,49]; Hexapoda [50,51,52,53,54], where mtDNA fragmentation was indeed suggested as an autapomorphy for the clade Mitodivisia [55]; Ichthyosporea [56,57]; and Saccharomycotina (e.g., [4,58]).

(ii) Chromosome architecture. Many examples are currently known of linear mtDNA [18,59]. Moreover, mtDNA is not always organized as a single chromosome; many species with multipartite mitochondrial genomes have been identified. Among Metazoans, linear chromosomes are known to be present in mammals with a wide array of concatenated forms ([60] and references therein); all medusozoans (cnidarians, excluding Anthozoa) analyzed so far show linear mtDNAs, which are further subdivided into multiple chromosomes in Hydrozoa and Cubozoa [40,42,43,61,62,63]. Linear, multipartite mtDNAs are also known to exist in calcareous sponges [13,40,41].

Among Fungi, the “broken-circle theory” has now been discontinued and the existence of polydisperse, linear mtDNAs in the brewer’s yeast *Saccharomyces cerevisiae* and in other yeasts is currently accepted [4,58,64,65,66,67]. It appears that linear mtDNA forms evolved from circular chromosomes in yeasts, but the shape of the genome also depends on the life stage of the yeast cell, with linear concatenamers dominating in mature bud cells [58,65,68]. More generally, in yeasts and land plants, mitochondria are best described as concatenated, linear-branched structures [15,26,29].

Among Alveolata, the ciliates *Paramecium* and *Tetrahymena* have been known, since 1968, to possess linear mtDNA [6,69,70,71]; the apicomplexan *Plasmodium* has a small 6 kb-long linear mtDNA with only three protein coding genes [72,73]. Additionally, mitochondria of *Amoebidium parasiticum* (Opisthokonta: Ichthyosporea) harbor several hundreds of small linear chromosomes [57].

Finally, besides the core mitochondrial genome, many land plant species and fungi harbor linear mitochondrial plasmids (e.g., [26,74,75] and references therein), which were reported from ciliates as well [76].

(iii) Genome size. Genome size is highly variable among eukaryotes, ranging from 6 kb in apicomplexan [23,24,72,73] and <13 kb in some green algae [77], ctenophores [78,79], and some fungi [80]; through 43 kb in placozoans [78,81] and >70 kb in choanoflagellates and ciliates [57,76]; up to >200 kb in other green algae and fungi [82,83,84], and 11 Mb in flowering plants [38]. Moreover, phenomena of the punctuated expansion of mtDNA have been reported within clades with generally reduced genome size (e.g., frogs [85], ark shells [86,87]). In most cases, this variability is not related to gene content; rather, the expansion and reduction of the intergenic region appear to be the main drivers of genome size among eukaryotes (e.g., [38,56,58,82,83,88,89]).
(iv) Gene content. Only three genes are located in the mtDNA of apicomplexans and their relatives [90,91,92], as well as in dinoflagellates [23,24]; only a dozen genes are encoded in euglenozoans’ mtDNAs [19], but up to ~100 have been identified in jakobids. The order Jakobida is included in the eukaryotic supergroup Discoba (see [93,94,95] and references therein); jakobids have been found to have up to ~100 mitochondrially-encoded genes [96], and, to the best of our knowledge, *Andalucia godoyi* has the most gene-rich mtDNA [97]. The choanoflagellate *Monosiga brevicollis* has an intermediate gene complement of 55 genes [57], while there are 47 for the ichthyosporean parasite *Sphaerothecum destruens* [98]. Conversely, a relatively constant gene content is known to be present in fungi and animals [56].

Chytridiomycetes typically harbor circular mtDNAs coding for the full complement of genes inferred from the opisthokont common ancestor, including tRNAs; mtDNAs from other Fungi appear to have lost many genes [56]. All yeast mtDNAs encode for three subunits of complex V (*atp6*, *atp8*, and *atp9*), for apocytochrome b (*cytb*), and for three subunits of complex IV (*cox1*, *cox2*, *cox3*) [99,100,101,102]. Additionally, seven subunits of complex I (*nad1*-*6* and *nad4L*) and two additional genes (*var1* and *rpm1*) may be present in fungal mtDNAs. However, complex I subunits were lost in the *Saccharomyces* group, while the *var1* gene was lost in the *Candida* group ([58] and references therein).

In bilaterian animals, the gene content encompasses two subunits of complex V (*atp6* and *atp8*), three subunits of complex IV (*cox1*, *cox2*, *cox3*), apocytochrome b (*cytb*), and seven subunits of complex I (*nad1*-*6* and *nad4L*) (e.g., [40,103,104]). Nonetheless, several exceptions have been observed. For instance, the *atp8* gene is often very divergent (e.g., [89,105,106]) and in some cases it has been claimed to be completely absent ([78,107,108,109,110,111,112,113]; also see [88,114,115]). Furthermore, many Open Reading Frames (ORFs) with no clear homology have been detected in many bilaterian lineages (e.g., [89,103,116,117,118,119,120,121]).

However, the picture of mtDNAs gets more confused among non-bilaterian animals, and many other ORFs have been identified (reviewed in [40]). Placozoans are considered to likely possess the mtDNA that is more similar to that of the metazoan common ancestor [81], which is a large, circular molecule with a full complement of tRNAs [122]. The number of tRNAs is variable among sponges, from 2 to 27, and *tatC* and *atp9* genes may be found [40]. A handful of tRNAs have been identified in cnidarians (e.g., [78,123]), where additional genes are present ([124] and references therein); similarly, many genes that are usually found in animal mtDNA are missing from that of ctenophores (tRNAs, *atp6*, *atp8*; [79,81,116,125]).

This summary of mitochondrial molecular structures and architectures certainly gives an idea of the stunning variability of these organellar genomes, which largely surpasses that of plastid trans-splicing phenomena [19,78,81,126]; the use of different genetic codes [13,127,128]; bewildering gene rearrangement [81,82,83,84,85,86,87,88,105,106,129,130,131]; and biparental and doubly uniparental inheritance [28,30,58,132,133,134,135,136].

In the present paper, we obtained from GenBank all the available complete mitochondrial genomes and used a slightly modified version of a recently published tool [137] to analyze the dataset. Exactly 40 years after the first complete mitochondrial sequence, we present a general description of our results; we also identify mitochondrial features typical of different taxa, aiming to provide a global overview of mitochondrial molecular diversity.

## 2. Materials and Methods

Mitochondrial genomes were mined from NCBI GenBank database (accessed on February 2021) using two different queries: “mitochondrion(title) AND complete(title) AND genome(title)” and “mitochondrial(title) AND DNA(title) AND complete(title) AND genome(title)”. In order to avoid unnecessary network load to the database (and machine time for subsequent analyses), overrepresented species were manually identified and relative records were excluded. Only one representative—or a few of them, depending on whether different populations were available—for each of the excluded species was manually selected and added to the automatically generated list (Supplementary Table S1).

A customized version of the HERMES tool [137] was used to analyze the dataset. The method involves the computation of several variables from annotated complete mitochondrial genomes. Variables are associated to gene content, nucleotide composition, phylogeny, and more. In fact, a HERMES analysis is typically carried out in a phylogenetic framework, which must be separately assessed. These metrics are usually summarized in a single number, the HERMES index, by means of a maximum likelihood factor analysis. For the present purpose, though, the HERMES index itself and variables stemming from a phylogenetic tree—such as AMIGA [137], root-to-tip distance, and maximum likelihood distance)—which were obviously not available in this context, were excluded. The following 11 variables are, therefore, considered and computed for each entry: length of the mtDNA, topology (linear or circular), number of annotated genes, absolute value of the Strand Usage skew (SU-skew; see [137] for definition), A+T content, AT-skew, GC-skew, CAI, percentage of Unassigned Regions (URs), UR-based A+T content, and UR-based median length.

Many NCBI hits were discarded as unsuitable for further analysis due to annotation errors/flaws or unsupported format. Some examples are entries with no annotation (raw sequences) or those with no annotated Coding Domain Sequences (CDSs). In six cases, it was possible to edit the minor details of annotations to include sequences that would have been otherwise excluded (Supplementary Table S2). Unfortunately, the HERMES approach has different constraints on a mtDNA annotation when carrying out the analyses. For example, at least one gene must be annotated to compute the UR proportion, and at least one CDS must be annotated to compute CAI. Consequently, our pipeline is blind to mitochondrial chromosomes where only tRNAs are annotated (or even no genes at all), which is sometimes the case for multipartite mtDNAs. It is also blind to unannotated entries resulting, for example, from studies on mitochondrial variation and displaying only mutations with respect to a reference sequence, and to entries with no sequence and linked to assembly data are also not detected.

The taxonomic information was retrieved from the NCBI page of each entry. We used the python class NCBITaxa from the package ETE Toolkit [138] to assign each lineage name to its proper taxonomic rank. All the analyses were carried out using custom-tailored Pyhton3 and R [139] scripts (available from F.P. and A.F. upon reasonable request). Plots were displayed using the “ggplot2” R package [140]. We calculated the Spearman’s rank correlation coefficient between pairwise groups of variables through the function rcorr from the “Hmisc” R package [141] and displayed the results through correlograms using the “corrplot” R package [142].

We used the database WoRMS [143] to collect ecological information such as feeding type and functional group. These pieces of information are annotated with a three-rank quality score; data marked with the lowest rank (“unreviewed”) were discarded from our analysis. Each piece of information was recorded along with the respective life stage. Ecological data were recorded for three taxonomic ranks: species, genus, and family. They were then applied to all matching entries using the package “worrms” [144].

## 3. Results and Discussion

### 3.1. Dataset Composition

**Figure 1:**
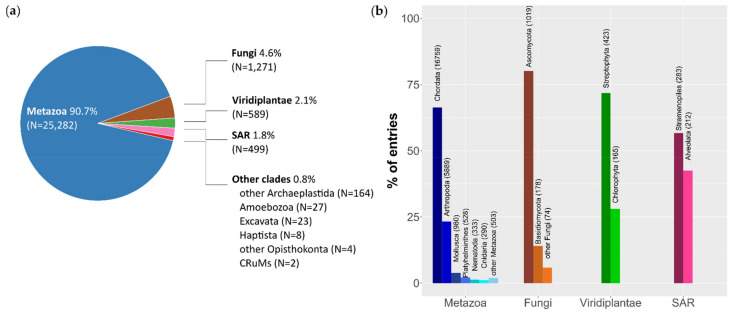
Database composition. (**a**) Major eukaryote subdivisions; (**b**) composition within the three major kingdoms (Metazoa, Fungi, and Viridiplantae) and the SAR clade.

The two queries combined, filtered for overrepresented taxa, returned 31,065 entries. Out of these entries, we were unable to compute the variables of 3192 entries because of poor annotation or unsupported format. Overall, we discarded 10.50% of the Metazoa entries (2965 entries), 11.90% of the Fungi entries (171 entries), 4.69% of the Viridiplantae entries (29 entries), and 3.56% of the remaining entries (27 entries). All entries were correctly assigned to one of the major eukaryotic subdivisions ([145]; Figure 1a): Diaphoretickes, comprised by Archaeplastida (including Viridiplantae), Excavata, Haptista, and SAR clade (Stramenopiles, Alveolata, Rhizaria); Amorphea, comprised by Amoebozoa and Opisthokonta (including Fungi and Metazoa); and CRuMs (Collodictyonidae, Rigifilida, Mantamonas). Only five GenBank entries were not correctly placed, four of which were eukaryotes *incertae sedis* (GenBank Accession Numbers NC_034794, NC_036491, MN082145, MG202007), while the last one was a dsDNA virus (GenBank Accession Number BK012062) that was removed from subsequent analyses.

The current version of the database was made publicly available as a CSV-formatted plain text file along with most R functions used for the present work on the MoZoo GitHub page, at the URL https://github.com/mozoo/almighto (accessed on: 7 July 2021). The final dataset is a 27873 × 80 matrix, where each row contains a taxonomic entry and columns are as follows:

1–2: accession number and definition of the genome on the NCBI page.

3–13: the 11 variables described above, which were obtained by the modified version of HERMES.

14–49: taxonomic ranks retrieved by NCBITaxa. The intersection of a row and a column is the entry’s lineage name for that taxonomic rank or NA if information is missing. In the 49th column, which is named “Eu_divisions”, each entry is placed in one of the major eukaryotic subdivisions described above.

50: the mitochondrial genetic code, which was retrieved from the relevant NCBI Taxonomy page.

51–80: ecological data. Each column is a different ecological feature. For the “functional group” columns, the value in each cell can be “FuncAdult” or “FuncLarva”, depending on which life stage the feature is at, or NA if information is missing. The same organization was used for the “feeding type” columns using the values “FeedAdult” and “FeedLarva”.

As expected, most of the retrieved entries come from the kingdom Metazoa (Figure 1a). Most of these entries belong to the phyla Chordata (66.2%) and Arthropoda (23.3%) (Figure 1b). On average, we obtained 2.4 mitogenomes per (available) species in Chordata, while the mean value for metazoans was 1.98 (1.44 for Arthropoda, 1.59 for Mollusca, 1.51 for Nematoda). In Fungi, most of the entries belong to the Ascomycota phylum (80.2%), which is mainly grouped in two classes, the Sordariomycetes and the Saccharomycetes, 41.1% and 36.1% of the Ascomycota entries, respectively (Figure 1b). On average, 3.13 entries correspond to each Sordariomycetes species, and 2.61 entries correspond to each Saccharomycetes species. Among Viridiplantae, the richest phylum is the Streptophyta, which is mainly represented by the classes Magnoliopsida (72.8%) and Bryopsida (11.3%) (Figure 1b). On average, 1.56 entries correspond to each Magnoliopsida species, whereas 1.28 entries correspond to each other Viridiplantae species.

The SAR clade is the third biggest clade in the dataset, after Opisthokonta and Archaeplastida (Figure 1a). It is divided into three main clades: Alveolata (42.5% of SAR entries), Stramenopiles (56.7% of SAR entries) and Rhizaria (4 entries) (Figure 1b). On average, 2.06 entries correspond to each Alveolata species; 1.73 entries correspond to each Stramenopiles species.

### 3.2. Mitogenome Reduction and Expansion

Excluding multipartite mtDNAs, the shortest complete mitogenomes in our dataset belong to three different Chinese isolates of the genus *Babesia*, an apicomplexan taxon that causes babesiosis, a tick-transmitted disease. Their linear mitogenome ranges from 5767 bp to 5790 bp and it encodes for nine genes: three protein coding genes and six rRNA genes [146]. The shortest metazoan mitogenome was the Ctenophora *Mnemiopsis leidyi*, which resulted in only 10326 pb long: this is mostly due to the absence of tRNA genes, to the scarcity of intergenic nucleotides, and to the relocation of atp6 to the nuclear genome [79].

On the contrary, the longest mitogenomes found belong to *Corchorus capsularis* and *Corchorus olitorius*, 1999 kbp and 1829 kbp, respectively (GenBank Accession Numbers NC_031359 and NC_031360, respectively). The latter species are commonly named jute and belong to the Malvaeae family. In Metazoa, the longest mitogenomes belong to ark shells of the genus *Anadara* (GenBank Accession Numbers NC_020787, NC_024927, KF750628); the mtDNA encodes for 42s tRNA and for a total of 56 genes, which constitute the largest number of tRNAs and genes encoded by a metazoan mtDNA. However, its length is mostly due to URs, which represent 67.7% of the entire sequence. The mtDNA was found to be 47–50 kbp long, depending upon the number of repeats in the URs [87]. However, if considering mitogenomes composed of several chromosomes, then the longest metazoan mitogenome belongs to the calcareous sponge *Clathrina clathrus* (six chromosomes, for a total length of 51 kb; [13]). 

**Figure 2:**
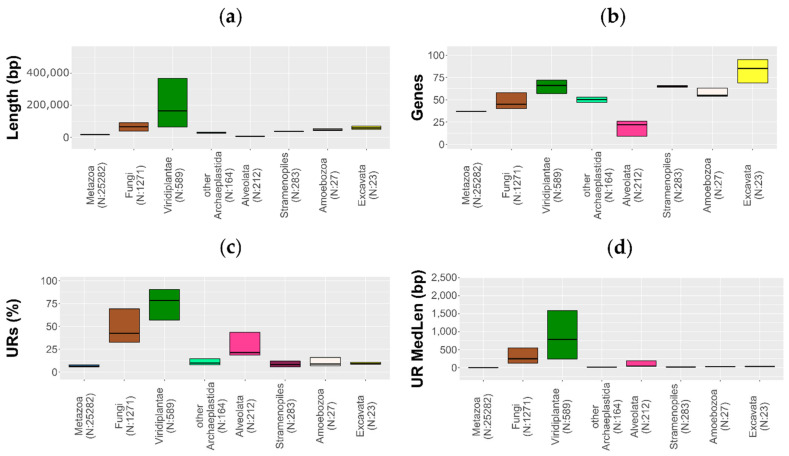
Mitochondrial genome dimension. The thick line depicts the median value; the boxplot ranges from the first to the third quantile. (**a**) mtDNA length (bp); (**b**) number of annotated genes; (**c**) UR content (%); (**d**) UR median length (bp).

**Figure 3:**
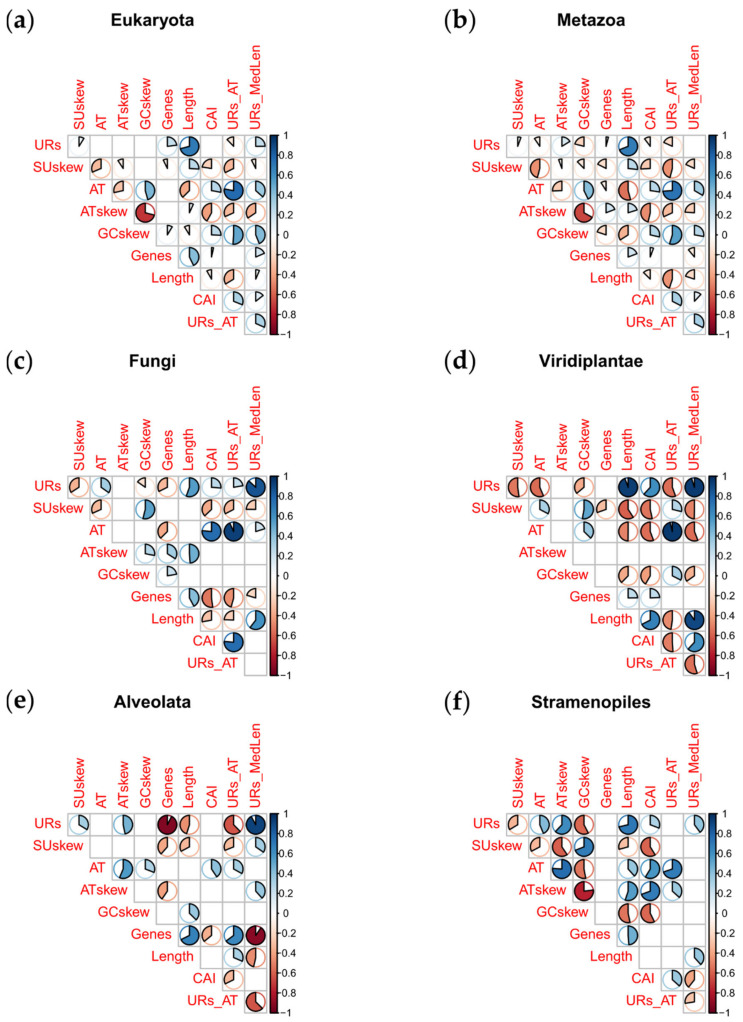
Correlograms for major mtDNA features. Each pie chart represents the value of a significant Spearman’s rho; where the pie chart is not shown, the correlation is not significant. A blue pie shows a positive Spearman’s rho, increasing clockwise from 0 to 1; a red pie shows a negative Spearman’s rho, increasing counterclockwise from 0 to 1. (**a**) Whole database; (**b**) Metazoa; (**c**) Fungi; (**d**) Viridiplantae; (**e**) Alveolata; (**f**) Stramenopiles.

Viridiplantae show the highest median in length, URs number, and URs median length among eukaryotes, as well as a high variability inside the clade (Figure 2a,c,d). Globally, the mtDNA length seems to increase with the UR content (Figure 3; Supplementary Figure S1). It is worth recalling that metazoans comprise the largest part of our dataset and may consistently drive the observed pattern; nonetheless, the correlation between mtDNA length and UR content was also observed in the isolated groups—Metazoa (Figure 3b), Fungi (Figure 3c), Viridiplantae (Figure 3d) and Stramenopiles (Figure 3f). However, in Alveolata (which includes apicomplexans), the length is negatively correlated with URs, but positively correlated with the number of genes (Figure 3e). Therefore, although metazoans share a reduced mtDNA with alveolates, in the latter group this reduction appears to be associated with gene loss rather than to URs reduction. Indeed, the Alveolata show the lowest median in length and genes, even if they show the third richest mtDNA in terms of URs (Figure 2a–c).

**Figure 4:**
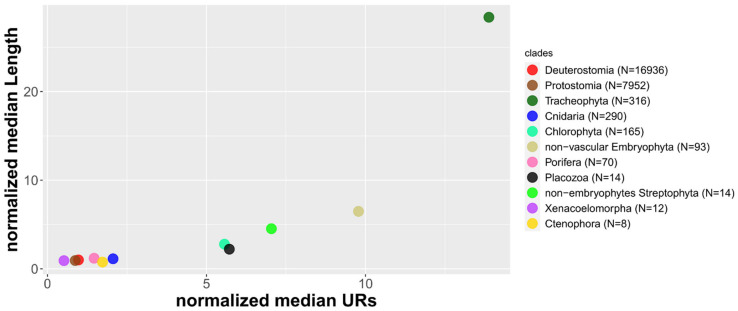
Mitogenomes expansion and contraction. For each group, the median UR content (%) has been normalized on the whole-database median UR content; the median length (bp) has also been normalized on the whole database median length.

It has been shown that the expansion of the mitochondrial genome is mostly associated with the expansion of non-coding or unassigned regions [56]. Among Viridiplantae, the mitogenome expansion is concurrent with the transition from water to land and it accelerated after the appearance of vascular plants [147,148,149]. Indeed, Chlorophyta shows the smallest mtDNA in terms of length and URs content. It is followed by the freshwater green algae in the Streptophyta (named non-embryophytes Streptophyta in Figure 4), the non-vascular Embryophyta (mosses, liverworts, and hornworts), and the Tracheophyta, which shows the longest and UR-richest mtDNA (Figure 4). This data underly an evolutionary pattern from the (hypothetical) ancestral mtDNA of Viridiplantae to the more derived and longer one of vascular plants. Conversely, among animals, an opposite autapomorphy seems to have arisen in Bilateria: mitogenomes from Porifera, Cnidaria, and Placozoa are generally regarded as more similar to the metazoan common ancestor, and on average, they are larger and harbor more unassigned regions (Figure 4; [150]).

The gene content is highly variable in eukaryotes, and during the evolution the mtDNA underwent losses and relocations of genes to the nucleus. Species in the Jakobida clade are considered the eukaryotes with the mtDNA most similar to the ancestral state, since they show a high gene content and some unique mitochondrial genes, such as the RNA polymerases [93,96]. Indeed, the clade Excavata, which includes the order Jackobida, shows the highest median gene content among eukaryotes (Figure 2b).

The protein-encoding genes are well conserved in the three main kingdoms: 14 in the Fungi, 13 in the Metazoa (excluding non-bilaterians), and 24 in the Viridiplantae [151]. The higher standard deviation of the gene content in Viridiplantae and Fungi (Figure 2b) is mainly due to the homing endonucleases encoded inside the introns and unassigned ORFs [82,152,153]. 

### 3.3. The Strand Asymmetry in Eukaryota

The Metazoa is the only clade showing a negative median for the GC-skew (Supplementary Figure S2), meaning that the cytidines are overabundant on the (putative) plus strand.

Moreover, AT-skew and GC-skew are strongly inversely correlated in Metazoa (Figure 3b), as well as in Stramenopiles (Figure 3f); on the other hand, the two variables are directly correlated in Fungi (Figure 3c), and have no significant correlation in Viridiplantae and Alveolata (Figure 3d,e). Therefore, in Metazoa, the plus strand is rich in A and C, whereas the minus strand is rich in T and G. According to the literature, this feature is due to the unidirectional replication of metazoan mitogenome. The H strand (which most of the times is considered the minus strand) is firstly replicated as single-stranded; during this condition, the deamination phenomenon is more frequent, leading to the mutation of C into U and A into hX (which base pairs with a C on the opposite strand [154]).

Different kind of correlations in the other kingdoms could be due to different replication and repair mechanisms of the mitogenome; in fact, a similar explanation has been proposed for the different evolutionary rates among eukaryote mtDNAs [155]. For instance, in Eubacteria, the GC-skew can be used to determine whether there are multiple origins of replication or not [156]. Although mitogenome replication is poorly understood outside metazoans, evidence suggests that plants, *Plasmodium falciparum*, and yeasts mitogenomes replicate through a rolling circle mechanism [157,158]. A thorough revision of mtDNA replication dynamics is well beyond the purpose of the present paper, and further investigation is needed to fully unveil and understand the different DNA replication mechanisms in different eukaryotic mitochondria, as well as to associate them to precise nucleotide compositional biases.

**Figure 5:**
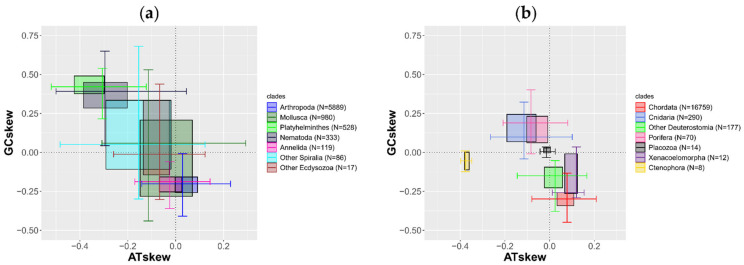
Bivariate boxplots of AT-skew and GC-skew. The lines within the boxplots depict the two median values; the boxplot ranges from the first to the third quantile along both axes; whiskers extend to roughly 95% confidence interval. For the sake of visibility, genomes have been divided into protostomes (**a**) and non-protostomes (**b**).

Notably, there are many exceptions even in Metazoa. In some cases, a reversed strand asymmetry (RSA) can be observed, so that on the plus strand there are more G than C as well as more T than A (resulting in a positive GC-skew and a negative AT-skew). This can be found, for example, in Porifera, Cnidaria, Platyhelminthes, Nemertea, and Nematoda, whereas in other spiralians, Mollusca, and other Ecdysozoa both conditions are present (Figure 5a,b). Moreover, the RSA can also be observed in other phyla at lower taxonomic ranks; examples are reported in the literature for fish [159], echinoderms [160], and arthropods [161,162]. The RSA is often related with the inversion of the control region; an AT-rich region is normally pivotal for the replication and determines which strand is replicated first [163,164]. Therefore, an inversion of the control region could invert the mutational pattern on both strands [162,165]. The localization of this region is not easy, and since it is one of the most variable regions of the mitogenome, it is impossible to align if the phylogenetic distance between the species is high [161]. Therefore, it is still uncertain if the inversion of the control region is the only process that can lead to RSA. Although some examples have been reported, a phylogenetically wider analysis is needed to determine what can affect the strand asymmetry.

### 3.4. Codon Adaptation and A+T Content

Mitogenomes are generally biased toward a high A+T content (which implies a low G+C content). Indeed, all the clades show a median above 50% (Supplementary Figure S2B). As mentioned before, the replication of the mtDNA leads to the deamination of A and C on the H strand. However, the deamination of C is more common; this results in an accumulation of T on the H strand, and of A on the L strand [166]. Therefore, the replication process may explain the biased A+T content as well as the opposite values of AT-skew and GC-skew, which are commonly observed, at least among Metazoa. However, as detailed above, the correlation between the skews is not consistent outside metazoans. Nonetheless, the A+T content is generally biased, with most clades showing an A+T content even higher than Metazoa (Supplementary Figure S2b). This data would imply that either the replication mechanisms outside metazoans lead to the increase in the A+T content without affecting the strand asymmetry or the nucleotide biases are affected by different causes, which are predominant in some clades and negligible in others. Viridiplantae showed the lowest A+T median content (55.8%), being even remarkably lower than that showed by the other Archaeplastida (70.4%) (Supplementary Figure S2b). It has been suggested that the A+T content decrease is concomitant with the land plants’ mtDNA genome expansion. The accumulation of URs can lead to a higher recombination frequency, which eventually raises the G+C content, thus decreasing the A+T content [147]. This mutational pattern would contrast with that observed in the Metazoa, where the replication leads to an accumulation of A and T, as reported above. The clades with a higher median URs and length show a lower A+T content (Table 1, Figure 4). The data agree with the hypothesis of Pedrola-Monfort and colleagues [147]; moreover, in Viridiplantae, URs and A+T content are negatively correlated, (Figure 3d). However, only non-vascular Embryophyta show a significant negative correlation (Table 1).

In Metazoa, the median A+T content is 60.3%; moreover, they show a high variability (Supplementary Figure S2B). Arthropoda and Nematoda show the highest median A+T content among metazoans at 76.4% and 74.1%, respectively. On the other hand, the lowest A+T content can be observed in the phylum Chordata (57.6%). 

The CAI statistic is a measure of how unbalanced the use of codons is in the same codon family. Their biased use can be explained in several ways. Specific codons can be selected to enhance translation efficiency; for instance, a correlation between the most used codons and the most abundant tRNA has been reported in bacteria [167,168], with the accepted idea being that the tRNA bias affects codon usage.

However, this does not seem the case for mtDNA, where codons seem to be biased according to the mitochondrial mutational pattern. More specifically, vertebrates A and C are found in the most frequent nucleotides at the third codon position [169], and in this clade most of the genes are located on the L strand, the one that in fact mostly accumulates A and C. In Bivalvia, where the strand asymmetry is reversed, the most used codons end with T and G [170]. Several works report that in Arthropoda and Nematoda mitogenomes, the most used codons end with A and T [171,172,173]. As detailed above, these phyla show the highest A+T content among Eukaryota; the neutral accumulation of A and T is confirmed by the highest frequency at the third codon position compared to the first and the second one [174]. Moreover, the accumulation of A and T is highly correlated with a biased use of codons inside Arthropoda and Nematoda (ρ = 0.79 and *p*-value = 0, ρ = 0.58 and *p*-value = 0, respectively). Indeed, comparisons between arthropod mtDNAs confirmed that mitogenomes richer in AT use NNT and NNA codons more frequently [175].

Interestingly, Actinopterygii show one of the lowest A+T content in Eukaryota (55.4%). In some entries, the AT-skew and the GC-skew show the same sign; moreover, in the cyprinid *Opsariichthys bidens*, it has been proven that C is the most used nucleotide at the third codon-position, while G-ending codons are more frequent than in other Chordata, suggesting that this feature is a result of a more efficient repair reaction to deamination [159]. This would also explain the low A+T content and the same sign of AT-skew and GC-skew in some Actinopterygii entries.

## 4. Conclusions and Final Remarks

Unfortunately, it was revealed that inconsistent annotation conventions led to systematic biases in data. Differences in the strand asymmetry can sometimes be related to different annotation decisions. Indeed, the signs of AT-skew and GC-skew depend on how the authors decide which strand is the plus strand, as a positive AT-skew on the L strand is obviously associated with an opposite negative AT-skew on the H strand. However, the localization of the L strand is not obvious for the clades where genes are located on both strands.

For example, we discovered that the order Unionida is split into two groups: 42 entries show RSA (as the other Bivalvia orders), while 119 entries show normal strand asymmetry (Supplementary Table S3). To prove that these groups are due to annotation issues, we calculated the number of genes on the plus strand for each entry, the number of genes on the minus strand, and on which strand the *cox1* gene is located, since its position is often taken as a reference to decide on the location of the plus strand. In the first group, most of the genes are located on the minus strand for all the entries, but the *cox1* gene is located on the plus strand; in the second group, most of the genes are located on the plus strand for all the entries, but the *cox1* gene is located on the minus strand (Supplementary Table S3). Although different gene orders have been detected in the order Unionida, the *cox1* gene in each group is located on the strand that harbors less genes [176]. Therefore, the authors applied different procedures to determine the plus strand: it is likely that the first group selected the strand with the *cox1* gene as the plus strand, whereas the second group selected the strand with more genes than the plus strand. This specific annotation issue has already been discussed [177]. Once again, we underline the necessity to determine and adopt conventions for the annotation of mitogenomes.

Moreover, when the two strands are correctly annotated, it is possible to unravel many phylogenetic artifacts. It has been proven that the RSA bias can affect the phylogenetic signal, also at the ammino acid level, thus leading to the clustering of clades that acquired RSA independently [161,178,179]. Therefore, before a phylomitogenomic analysis starts, it is pivotal to determine the strand asymmetry of each marker to exclude wrong phylogenetic signals.

To our knowledge, the database set up for the present paper is the first attempt to present most of the available mitochondrial genomes together. It has the potential to elucidate several molecular patterns underlying the figure of mitochondrial evolution across the eukaryotic domain. Further research is requested to overcome annotation errors and issues, and a release of the database including multipartite mtDNAs, as well as other GenBank entries, is currently under preparation in our laboratory. Recalling the vagaries of mitochondrial evolutionary history, it is clear that only an adequate sampling of eukaryotic biodiversity and the analysis of a huge number of genomes can shed light on the structure, architecture, and the role of the mitochondrial genome in the eukaryotic cell.

## Figures and Tables

**Table 1:** Mitochondrial genome expansion of land plants. The three columns present the A+T content percentage, the Spearman’s rho coefficient of correlation between A+T content and UR percentage on the mtDNA, and the *p*-value associated with the Spearman’s rho.

	A+T Content	Spearman’s Rho	*p*-Value
Chlorophyta	62.8%	ρ = 0.15	*p* = 0.0506
Non-embryophytes Streptophyta	60.3%	ρ = −0.51	*p* = 0.065
Non-vascular Embryophyta	58.9%	ρ = −0.64	*p* = 0
Tracheophyta	55.0%	ρ = 0.07	*p* = 0.2

## Data Availability

The database which is presented in the present manuscript is publicly available on the MoZoo GitHub page, at the URL https://github.com/mozoo/almighto (accessed on: 7 July 2021).

## Supplementary Materials

The following are available online at https://www.mdpi.com/article/10.3390/life11070663/s1, Figure S1: Correlograms for major mtDNA features, Figure S2: Nucleotide composition and strand asymmetry, Table S1: Manually excluded species and relative selected GenBank Accession Numbers, Table S2: Manually edited annotations, Table S3: Annotation bias in Unionida.
