# Supplementary material for: Mitochondrial Genomic Landscape: A Portrait of the Mitochondrial Genome 40 Years after the First Complete Sequence
Source: Life (Basel). 2021 Jul 6;11(7):663. doi: 10.3390/life11070663 (PMC8303319; doi:10.3390/life11070663)
Supplement: Supplementary file 1 [file life-11-00663-s001.zip › life-1281005-supplementary.pdf]

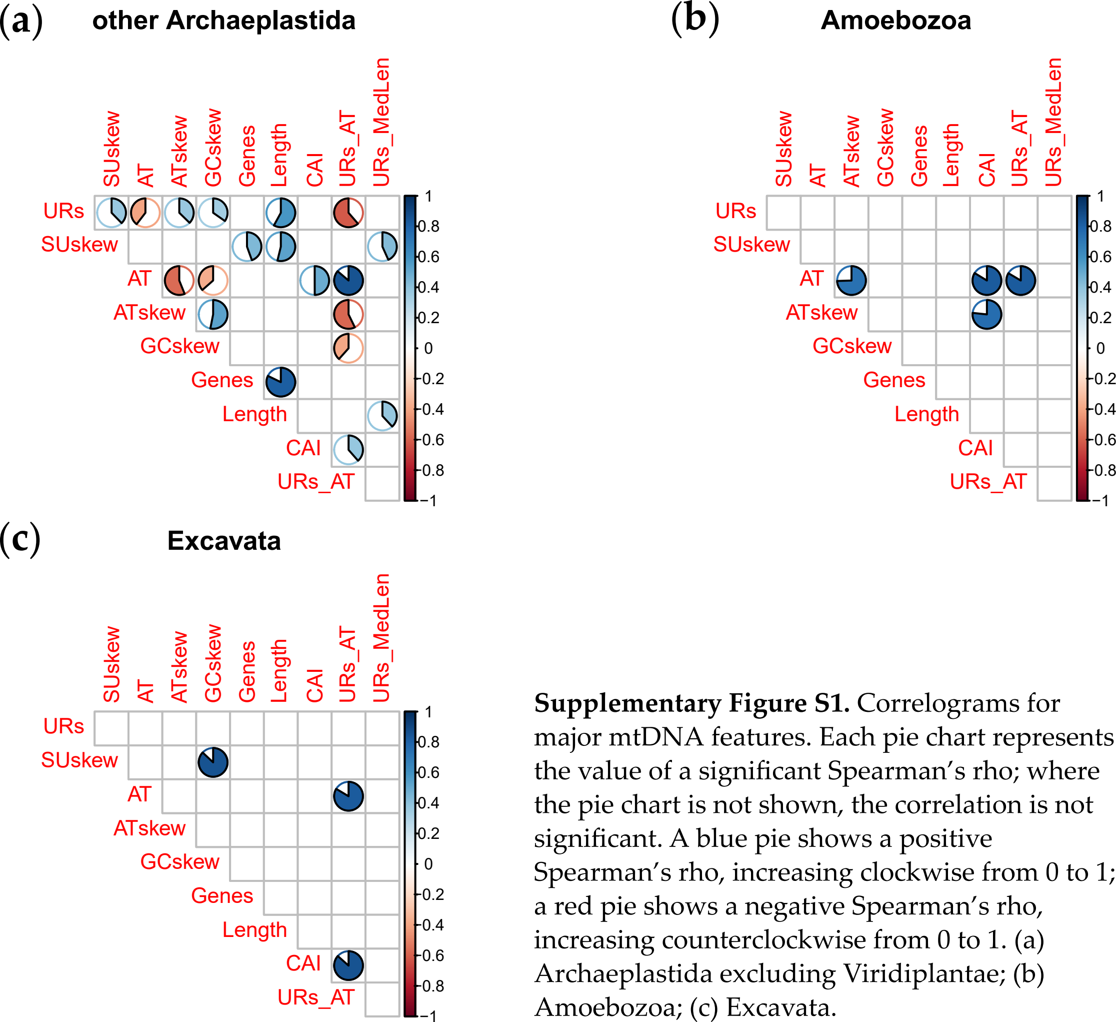

Supplementary Figure S1. Correlograms for major mtDNA features. Each pie chart represents the value of a significant Spearman's rho; where the pie chart is not shown, the correlation is not significant. A blue pie shows a positive Spearman's rho, increasing clockwise from 0 to 1; a red pie shows a negative Spearman's rho, increasing counterclockwise from 0 to 1. (a) Archaeplastida excluding Viridiplantae; (b) Amoebozoa; (c) Excavata.

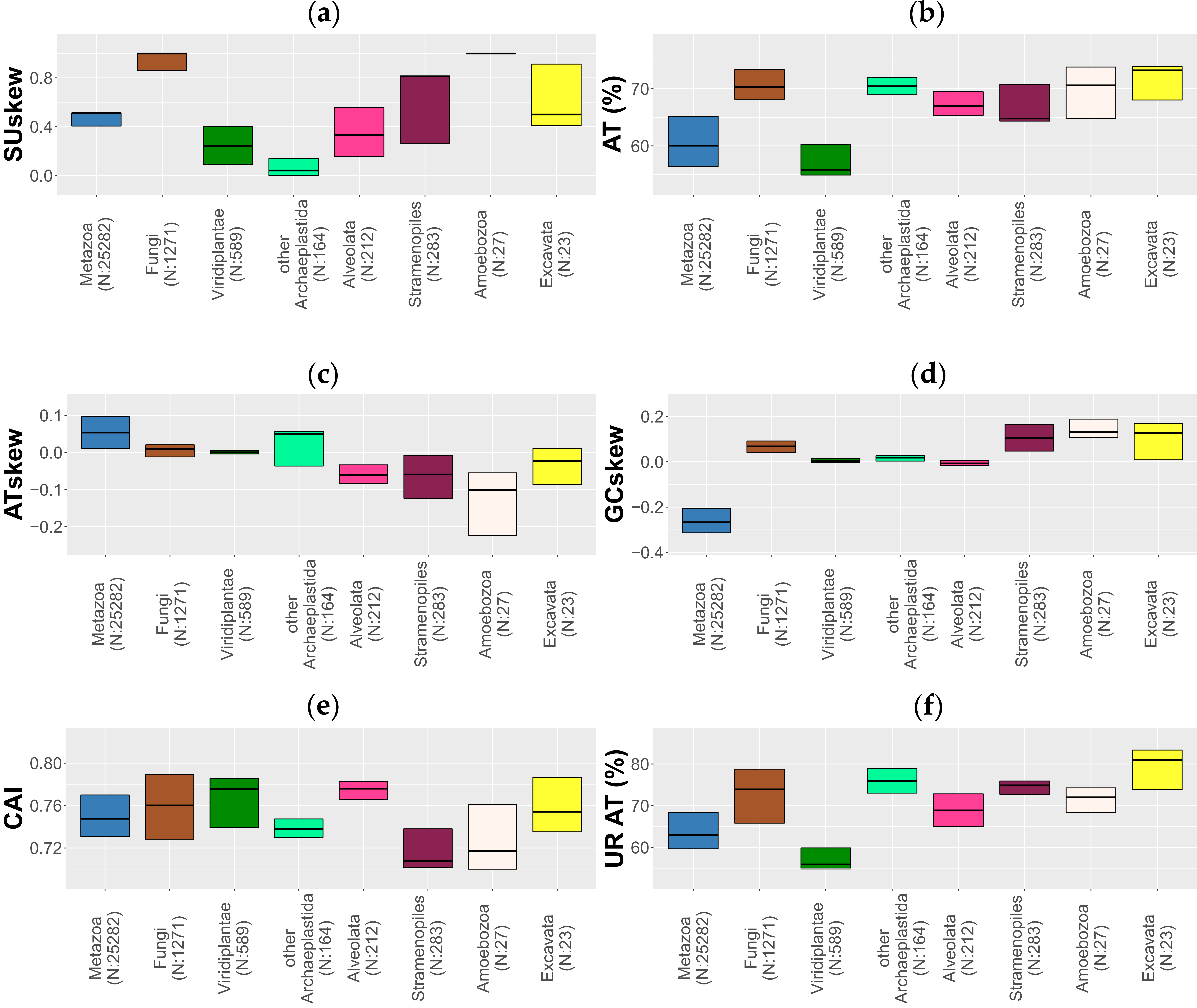

Supplementary Figure S2. Nucleotide composition and strand asymmetry. The thick line depicts the median value; the boxplot ranges from the first to the third quantile. (a) SU-skew; (b) AT (%); (c) AT-skew (%); (d) GC- skew; (e) CAI; (f) UR AT (%).

Supplementary Table S1. Manually excluded species and relative selected GenBank Accession Numbers.

Species	Phylum	Selected ID
Anopheles gambiae	Arthropoda	MG753766.1
Balaenoptera physalus	Chordata	MT410921.1
Bos taurus	Chordata	MN714217.1
Bubalus bubalis	Chordata	KX758298.1
Canis lupus	Chordata	AB499825.1
Chrysomya megacephala	Arthropoda	MH778860.1
Equus caballus	Chordata	KT221835.1
Fusarium asiaticum	Ascomycota	MN935318.1
Gallus gallus	Chordata	KY039386.1
Glyphis glyphis	Chordata	NC_021768.2
Homo sapiens	Chordata	KY411469.1
Homo sapiens neanderthalensis	Chordata	MG025540.1
Lycorma delicatula	Arthropoda	MT079445.1
Lynx pardinus	Chordata	KX911409.1
Menidia menidia	Chordata	MH538842.1
Mus musculus	Chordata	NC_005089.1
Ovis aries	Chordata	MT768219.1
Pan troglodytes	Chordata	NC_001643.1
Plasmodium falciparum	Apicomplexa	KY923536.1
Plasmodium vivax	Apicomplexa	KY923386.1
Spadella cephaloptera	Chaetognatha	KP899748.1
Spadella cephaloptera	Chaetognatha	KP899749.1
Spadella cephaloptera	Chaetognatha	KP899750.1
Spadella cephaloptera	Chaetognatha	KP899751.1
Spadella cephaloptera	Chaetognatha	KP899752.1
Spadella cephaloptera	Chaetognatha	NC_006386.1
Sus scrofa	Chordata	KX982659.1

Supplementary Table S2. Manually edited annotations.

Species	ID	Issue	Fix
Argopecten irradians	KT161259	The *rrnL* gene is annotated on the whole genome, resulting in no UR	Correct the annotation to "`14940..16286`"
Bugula neritina	NC_010197	A tRNA is annotated also on the whole genome, resulting in no UR	Remove the wrong annotation line
Ictalurus pricei	KJ496299	The *rrnL* gene is annotated on the whole genome, resulting in no UR	Correct the annotation to "`1986..3645`"
Placozoa sp.	NC_008832	No CDS annotation in version 4	Switch to version 3
Placozoa sp.	NC_008833	No CDS annotation in version 4	Switch to version 3
Placozoa sp.	NC_008834	No CDS annotation in version 4	Switch to version 3

Supplementary Table S3. Annotation bias in Unionida. Genes+, number of genes annotated on the plus strand; Genes−, genes annotated on the minus strand; *cox1* strand, strand where the *cox1* gene is annotated.

Species	ID	Genes+	Genes−	*cox1* strand	AT-skew
Aculamprotula tientsinensis	NC_029210	27	11	−	>0
Anodonta anatina	KF030962	26	12	−	>0
Anodonta anatina	KF030963	26	12	−	>0
Anodonta anatina	KF030965	27	11	−	>0
Anodonta anatina	KF030966	27	11	−	>0
Anodonta anatina	KF030967	27	11	−	>0
Anodonta anatina	KF030968	27	11	−	>0
Anodonta anatina	NC_022803	27	11	−	>0
Anodonta arcaeformis	NC_026674	26	11	−	>0
Anodonta cygnea	MF781083	26	11	−	>0
Anodonta cygnea	NC_036488	26	11	−	>0
Anodonta lucida	NC_026673	26	11	−	>0
Beringiana fukuharai	LC592401	26	11	−	>0
Beringiana fukuharai	LC592402	26	11	−	>0
Beringiana fukuharai	LC592403	26	11	−	>0
Beringiana fukuharai	LC592408	26	11	−	>0
Beringiana fukuharai	LC592410	26	11	−	>0
Cristaria plicata	GU944476	26	11	−	>0
Cristaria plicata	KM233451	25	12	−	>0
Cristaria plicata	NC_012716	25	11	−	>0
Cumberlandia monodonta	KU873124	26	12	−	>0
Cumberlandia monodonta	NC_034846	28	11	−	>0
Echyridella menziesii	KU873122	26	12	−	>0
Echyridella menziesii	NC_034845	27	11	−	>0
Elliptio complanata	BK010477	14	8	−	>0
Gibbosula crassa	NC_037942	27	11	−	>0
Hyriopsis cumingii	HM347668	26	11	−	>0
Hyriopsis cumingii	KM393224	26	11	−	>0
Hyriopsis cumingii	LC498621	26	11	−	>0
Hyriopsis cumingii	NC_011763	26	11	−	>0
Lamprotula coreana	NC_026035	26	11	−	>0
Lamprotula gottschei	KJ627225	26	11	−	>0
Lamprotula gottschei	NC_023806	8	29	−	>0
Lamprotula leaii	KC847114	26	11	−	>0
Lamprotula leaii	NC_023346	26	11	−	>0

Lamprotula tortuosa	KC441487	26	11	−	>0
Lamprotula tortuosa	NC_021404	26	11	−	>0
Lampsilis cardium	BK010478	26	11	−	>0
Lampsilis ornata	NC_005335	26	11	−	>0
Lampsilis powellii	MF326972	26	12	−	>0
Lampsilis powellii	NC_037720	26	12	−	>0
Lampsilis siliquoidea	MF326974	26	12	−	>0
Lampsilis siliquoidea	NC_037721	26	12	−	>0
Lanceolaria gladiola	KY067441	26	11	−	>0
Lanceolaria lanceolata	KJ775864	26	11	−	>0
Lanceolaria lanceolata	NC_023955	26	11	−	>0
Lasmigona compressa	NC_015481	28	10	−	>0
Leaunio lienosus	BK010479	23	11	−	>0
Lepidodesma languilati	NC_029491	26	11	−	>0
Margaritifera dahurica	NC_023942	27	11	−	>0
Margaritifera falcata	NC_015476	28	10	−	>0
Margaritifera margaritifera	BK010445	26	11	−	>0
Mutela dubia	NC_034844	26	11	−	>0
Nodularia douglasiae	KP970613	26	11	−	>0
Nodularia douglasiae	LC496352	25	12	−	>0
Nodularia douglasiae	LC589171	26	11	−	>0
Nodularia douglasiae	LC589172	26	11	−	>0
Nodularia douglasiae	MF314443	25	12	−	>0
Nodularia douglasiae	MT764726	26	11	−	>0
Nodularia douglasiae	MT955592	25	12	−	>0
Nodularia douglasiae	NC_026111	26	11	−	>0
Parvasolenaia rivularis	KY007142	26	11	−	>0
Parvasolenaia rivularis	NC_039839	26	11	−	>0
Potamilus alatus	KU559011	29	9	−	>0
Potamilus alatus	NC_033858	27	11	−	>0
Potamilus leptodon	NC_028522	26	11	−	>0
Potamilus streckersoni	MW413895	27	11	−	>0
Pronodularia japanensis	LC505454	26	11	−	>0
Ptychorhynchus pfisteri	KY067440	26	11	−	>0
Pyganodon grandis	FJ809755	26	11	−	>0
Pyganodon grandis	NC_013661	26	11	−	>0
Quadrula quadrula	FJ809751	26	11	−	>0
Quadrula quadrula	NC_013658	26	11	−	>0
Sinanodonta tumens	LC592406	26	11	−	>0
Sinanodonta woodiana	HQ283344	27	11	−	>0

Sinanodonta woodiana	HQ283345	27	11	−	>0
Sinanodonta woodiana	HQ283346	27	11	−	>0
Sinanodonta woodiana	HQ283347	27	11	−	>0
Sinanodonta woodiana	HQ283348	27	11	−	>0
Sinanodonta woodiana	MH349356	26	14	−	>0
Sinanodonta woodiana	MH349359	26	14	NA	>0
Sinanodonta woodiana	NC_024943	27	11	−	>0
Sinohyriopsis schlegelii	AP018550	26	11	−	>0
Sinohyriopsis schlegelii	AP018551	26	11	−	>0
Sinohyriopsis schlegelii	HQ641407	26	11	−	>0
Sinohyriopsis schlegelii	LC498622	26	11	−	>0
Sinohyriopsis schlegelii	NC_015110	26	11	−	>0
Solenaia carinata	KC848655	26	12	−	>0
Solenaia carinata	NC_023250	27	11	−	>0
Solenaia oleivora	KY007143	26	11	−	>0
Solenaia oleivora	MT477834	27	10	−	>0
Solenaia oleivora	NC_022701	26	11	−	>0
Toxolasma parvum	NC_015483	27	11	−	>0
Unio crassus	KY290447	26	12	NA	>0
Unio crassus	KY290448	26	12	NA	>0
Unio crassus	KY290449	26	12	NA	>0
Unio crassus	KY290450	26	12	NA	>0
Unio crassus	NC_033976	26	12	NA	>0
Unio delphinus	KT326918	26	12	−	>0
Unio delphinus	NC_033854	27	11	−	>0
Unio pictorum	HM014131	26	11	−	>0
Unio pictorum	HM014132	26	11	−	>0
Unio pictorum	HM014133	26	11	−	>0
Unio pictorum	HM014134	26	11	−	>0
Unio pictorum	MH349357	26	12	NA	>0
Unio pictorum	MH349358	26	12	NA	>0
Unio pictorum	NC_015310	26	11	−	>0
Unio tumidus	KY021074	26	12	−	>0
Unio tumidus	KY021075	26	12	−	>0
Unio tumidus	KY021076	27	11	−	>0
Unio tumidus	KY021077	27	11	−	>0
Unio tumidus	KY021078	27	11	−	>0
Unio tumidus	NC_033977	26	12	−	>0
Uniomerus tetralasmus	BK010480	23	10	−	>0
Utterbackia imbecillis	NC_015479	27	11	−	>0

Utterbackia peninsularis	HM856636	28	10	−	>0
Utterbackia peninsularis	NC_015477	26	12	−	>0
Venustaconcha ellipsiformis	FJ809753	26	11	−	>0
Venustaconcha ellipsiformis	NC_013659	27	10	−	>0
Aculamprotula scripta	NC_045529	11	26	+	<0
Acuticosta chinensis	MF687347	11	27	+	<0
Acuticosta chinensis	NC_042472	11	26	+	<0
Alasmidonta heterodon	NC_037431	11	27	+	<0
Alasmidonta varicosa	NC_038155	11	27	+	<0
Amblema plicata	NC_050056	11	26	+	<0
Anemina euscaphys	NC_026792	11	26	+	<0
Anodonta anatina	MN594531	11	26	+	<0
Anodonta cygnea	MN594532	11	26	+	<0
Anodonta exulcerata	MN594533	11	26	+	<0
Anodonta nuttalliana	MN594534	11	26	+	<0
Chamberlainia hainesiana	MK994771	11	26	+	<0
Chamberlainia hainesiana	NC_044110	11	26	+	<0
Cuneopsis capitata	NC_042469	11	26	+	<0
Cuneopsis heudei	MF687348	11	27	+	<0
Cuneopsis heudei	NC_042471	11	26	+	<0
Cuneopsis pisciculus	NC_026306	11	26	+	<0
Lamprotula caveata	KX091842	11	26	+	<0
Lamprotula caveata	NC_030336	12	25	+	<0
Lamprotula scripta	NC_030258	13	25	+	<0
Lanceolaria grayii	NC_026686	13	24	+	<0
Margaritifera margaritifera	MK421957	11	26	+	<0
Margaritifera margaritifera	MK421958	11	26	+	<0
Margaritifera margaritifera	MK421959	11	26	+	<0
Margaritifera margaritifera	NC_043836	11	26	+	<0
Microcondylaea bonellii	MK994773	11	26	+	<0
Microcondylaea bonellii	NC_044111	11	26	+	<0
Monodontina vondembuschiana	NC_044112	11	26	+	<0
Pilsbryoconcha exilis	MK994777	11	26	+	<0
Pilsbryoconcha exilis	NC_044124	11	26	+	<0
Pleurobema oviforme	NC_050057	11	26	+	<0
Popenaias popeii	NC_050058	11	26	+	<0
Potomida littoralis	KT247375	11	26	+	<0
Potomida littoralis	NC_030073	11	26	+	<0
Pseudanodonta complanata	MN594535	11	26	+	<0
Pseudunio marocanus	KY131954	11	26	+	<0

Pseudunio marocanus	NC_034911	11	26	+	<0
Schistodesmus lampreyanus	NC_042470	11	26	+	<0
Sinanodonta woodiana	KM434235	11	26	+	<0
Sinanodonta woodiana	MN594536	11	26	+	<0
Unio elongatulus	MN594537	11	26	+	<0
Unio mancus	MN594538	11	26	+	<0
